# Fiber-Optic Thermal Sensor for TiN Film Crack Monitoring

**DOI:** 10.3390/ma10111297

**Published:** 2017-11-11

**Authors:** Hsiang-Chang Hsu, Tso-Sheng Hsieh, Yi-Chian Chen, Hung-En Chen, Liren Tsai, Chia-Chin Chiang

**Affiliations:** 1 Department of Mechanical Engineering, National Kaohsiung University of Applied Sciences, Kaohsiung 807, Taiwan; gn1204774@gmail.com (H.-C.H.); al11062000@gmail.com (Y.-C.C.); liren@kuas.edu.tw (L.T.); 2 Department of Industrial Engineering and Management, Fortune Institute of Technology, Kaohsiung 831, Taiwan; srcx2s904@gmail.com; 3 Department of Mechanical Engineering, Wu Feng University, Chiayi County 62153, Taiwan; 2954883@gmail.com

**Keywords:** fiber Bragg grating (FBG), temperature sensitivity, TiN-coated FBG sensor, refractive index sensitivity

## Abstract

The study focuses on the thermal and temperature sensitivity behavior of an optical fiber sensor device. In this article, a titanium nitride (TiN)-coated fiber Bragg grating (FBG) sensor fabricated using an ion beam sputtering system was investigated. The reflection spectra of the FBG sensor were tested using R-soft optical software to simulate the refractive index sensitivity. In these experiments, the temperature sensitivity of the TiN FBG was measured at temperatures ranging from 100 to 500 °C using an optical spectrum analyzer (OSA). The results showed that the temperature sensitivity of the proposed TiN FBG sensor reached 12.8 pm/°C for the temperature range of 100 to 300 °C and 20.8 pm/°C for the temperature range of 300 to 500 °C. Additionally, we found that the produced oxidation at temperatures of 400–500 °C caused a crack, with the crack becoming more and more obvious at higher and higher temperatures.

## 1. Introduction

The safety-related resistance temperature detectors (RTDs) used in nuclear power plants (NPP) require fast dynamic performance [1]. In order to achieve dynamic performance sensor monitoring and diagnostics, the associated response times can be estimated in situ by noise analysis techniques [2]. RTDs are sensors that are used to measure temperature by directly registering electrical resistance [3], the resistive value of which changes simultaneously with temperature changes, and these sensors can be used to measure wide ranges of temperature, including temperatures from −50 to 500 °C for the thin film variety and temperatures from −200 to 850 °C for the wire-wound variety [4]. When using RTDs, the biggest of which are self-heating, the test current could result in measurement inaccuracy. Optic fibers are typically small in size, passive, immune to electromagnetic interference, resistant to harsh environments, and are capable of performing distributed sensing [5,6,7]. In a study conducted by Rajini-Kumar et al. [8], different types of metal-coated FBGs were tested for their temperature characteristics, and a CrN-coated FBG sensor exhibited a 14.0 pm/°C greater temperature sensitivity than a bare FBG [9]. Relatedly, in studies by the Zhang group regarding the design and analyses of periodic dielectric Bragg grating and associated waveguide temperatures [10,11], germanium-oxygen (Ge-O) bonds were found to play an important role during the index change process of FBG fabrication.

The mathematical modeling of fiber grating is generally accomplished using the coupled mode theory of wave propagation and the transfer matrix method [12,13,14]. Chiang et al. conducted a simulation study of coated FBG used as a temperature sensor using the GratingMOD of the RSoft Photonics computer-aided design (CAD) Suite (version 2016.19, Synopsys, Mountain View, CA, USA), according to the literature, where *α* was the thermal expansion coefficient of silica and was equal to 0.55 × 10^−6^, while *ξ* was the thermo-optic coefficient of the fiber material and was equal to 7.18 × 10^−6^ for photosensitive (PS) 1250/1500 (Fibercore) fiber [15].

The present study sought to develop a simple method for making a temperature model of TiN-coated FBG, and to study the role of such metal coating in promoting the temperature sensitivity and the crack characteristics of TiN-coated film of FBG in high-temperature environments. In this study, the TiN-coated FBG were analyzed applied to monitoring the temperature in the environment sensor. An experimental metal coating was developed, and the TiN FBG sensor was investigated with regard to its temperature sensitivity and crack characteristics. The measurement results, such as strain rate and other information, were detected by the response of the reflected spectrum of the TiN FBG. The results show that the TiN FBG sensor has a better sensing capability and monitoring response at higher temperatures than the bare FBG.

## 2. Working Principle of TiN-Coated FBG Temperature Sensors 

### 2.1. Fabrication of TiN-Coated FBG Temperature Sensors

In this study, the fiber studied was procured from commercial sources, was specially fabricated, and consisted of boron/germanium co-doped fiber (fiber type: photosensitive) 1250/1500, inner core diameter of 9.6 μm, and outer glass (SiO_2_) cladding diameter of 124.9 μm, supplied by Fibercore Ltd. (Southampton, UK). Bragg grating was written into the fiber by using a pulsed KrF-excimer laser at 248 nm wavelength (Coherent Xantos XS, 248 nm wavelength, 12 mJ/cm^2^, Coherent, Palo Alto, CA, USA). The grating region of the optical fiber was 5 mm in length, and was etched to a diameter of 60 μm in a buffered oxide etchant (BOE) solution, and then placed and fixed on a holder in order to be accurately sputtered.

**Figure 1:**
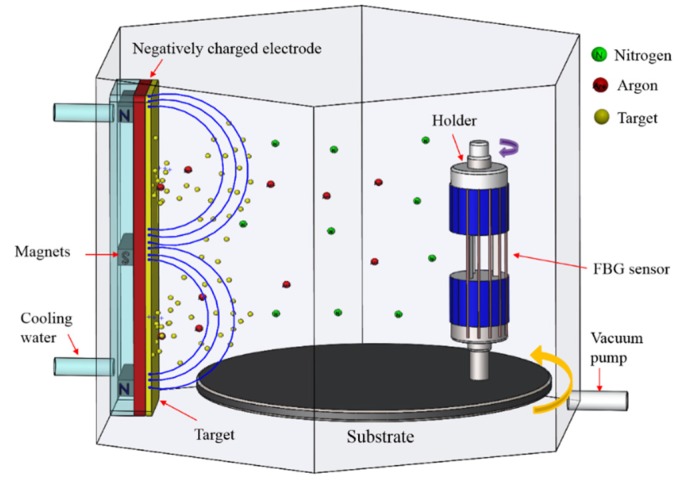
Rotating the FBG sensor holder in the vacuum chamber.

In this sputtering, a form of physical vapor deposition (PVD), the material was deposited with high energy ions from a plasma. Argon (Ar) was used to facilitate the deposition and did not react with the deposited material. The metal thin film was coated onto the FBG, and its reaction with nitrogen gas caused the formation of TiN. An ion beam sputtering system was used to deposit a uniform TiN film by rotating the substrate and holder in a vacuum chamber, as illustrated in Figure 1. Pre-sputtering was first conducted for 30 min to eliminate any vacuum chamber contamination. The Ar to N_2_ gas ratio was 80:20. The sputtering process pressure of the system was 6 × 10^−3^ Torr, and the power of the Ti target material was 70 W. The film was deposited for 15 min to approximate a film thickness of 0.82 μm.

**Figure 2:**
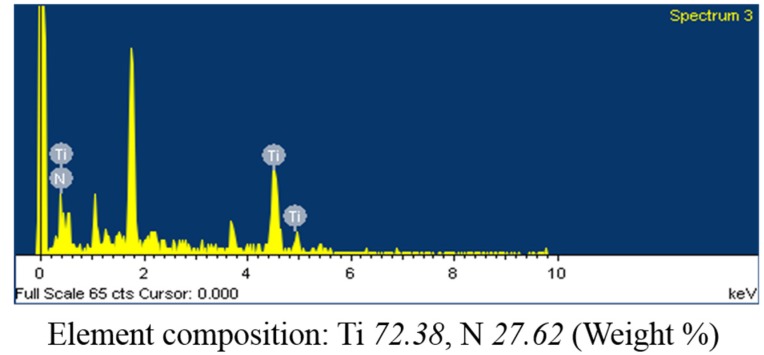
EDS analysis results of the TiN-coated FBG sensor.

**Figure 3:**
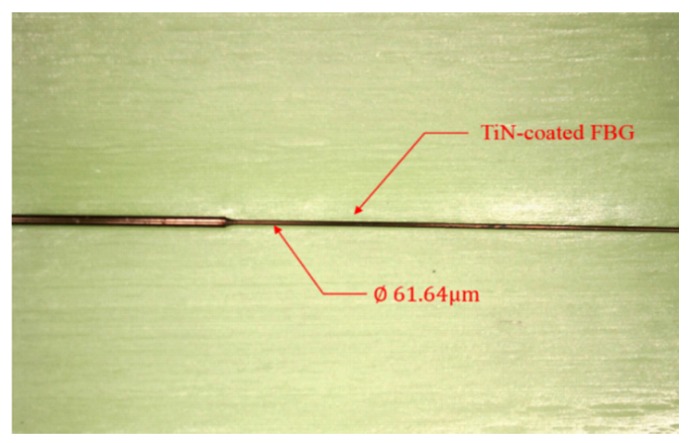
Photographic view of TiN-coated FBG.

The composition of the TiN film was characterized by a scanning electron microscope equipped with energy dispersive spectroscopy (EDS). Figure 2 shows a spectrogram of the EDS analysis results of the TiN-coated FBG sensor, which indicate that titanium and nitrogen were detected on the FBG. The Ti content was about 72.38 wt %, and the N content was about 27.62 wt %. The FBG and the TiN film coating are shown in Figure 3.

### 2.2. Principle of TiN-Coated FBG Temperature Sensors

The FBG can be characterized by its Bragg wavelength, which is the reflected wavelength of light from the grating. The Bragg wavelength is $\lambda_{B}=2n_{eff}$, where $n_{eff}$ is the effective refractive index of the fiber core and **Λ** is the grating period.

If the temperature is equal to zero, the Bragg wavelength of bare FBG sensor is given by

(1) $$\frac{\Delta \lambda_{B}}{\lambda_{B}}=\left[\alpha_{s}+\xi \right]\Delta T$$

where **Δ*T*** = ***T*** − ***T*_0_** is the change in temperature, $\alpha_{s}$ is the thermal expansion coefficient of silica equal to 0.55 × 10^−6^, and $\xi =\left(1/n_{eff}\right)\left(\partial n_{eff}/\partial T\right)$ is the thermo-optic coefficient of the fiber equal to 7.18 × 10^−6^ [15].

According to the literature [9,16], for a temperature change **Δ*T***, the thermal expansion coefficients and the refractive index can be defined as

(2) $$\frac{\Delta \lambda_{B}}{\lambda_{B}}=\left[\alpha_{s}+\left(1-P_{e}\right)\left(\alpha_{TiN}-\alpha_{S}\right)\eta +\xi \right]\Delta T$$

(3) $$\eta =\left[\left(\frac{A_{TiN}}{A_{S}}\right)\left(E_{TiN}\right)\right]/\left[\left(\frac{A_{TiN}}{A_{S}}\right)\left(E_{TiN}\right)+E_{S}\right]$$

where $P_{e}$ is the photoelastic constant of the fiber and $\alpha_{TiN}$ is the thermal expansion coefficient of TiN material, $E_{TiN}$ and $E_{S}$ are the Young’s moduli of the TiN material and the silica glass, respectively, and $A_{TiN}$ and $A_{S}$ are the cross-sectional areas of the TiN material and the silica glass, respectively.

For FBG coated with TiN, a change in the temperature causes a change in the grating period due to the thermal expansion of the fiber and the strain induced by thermal expansion of the TiN coating material. In addition, the refractive index of the core changes due to the thermo-optic effect. The temperature sensitivity of TiN-coated FBG is given by

(4) $$\frac{\Delta \lambda_{B}}{\lambda_{B}}=\left[\left(1-P_{e}\right)\alpha_{TiN}+\xi \right]\Delta T$$

where $P_{e}$ is the photoelastic constant of the fiber and $\alpha_{TiN}$ is the thermal expansion coefficient of the TiN material. The theoretical temperature sensitivity can thus be determined by calculating that of the metal film combined with the FBG. The metal film deposition technique can be effectively utilized to deposit TiN coating, and by adding this coating, the temperature sensitivity of the FBG sensor in high temperature environments was increased.

## 3. Experiment

### 3.1. Measurement of TiN-Coated FBG Sensor

**Figure 4:**
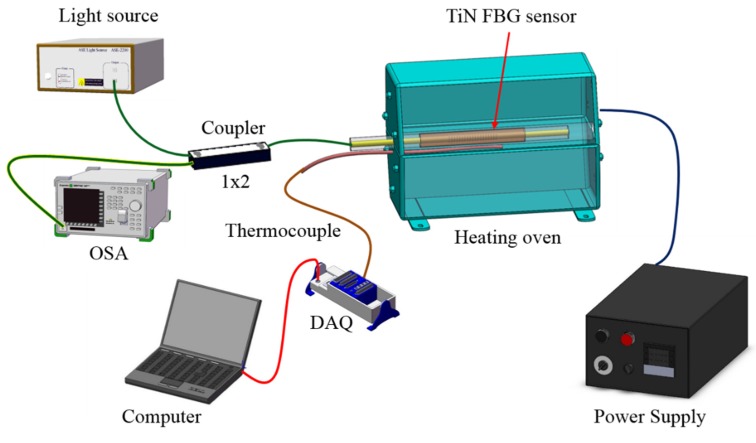
The experimental setup of the temperature sensing test.

The high temperature sensor was fabricated by using a DC magnetron to sputter physical vapor deposition (PVD) TiN onto the surface cladding of the FBG sensor. The resulting novel metal-coated nitrogen-doped high temperature sensor was then studied using the experimental framework illustrated in Figure 4. A broadband light ray was injected using a super luminescent diode (SLD, DL-BP1-1501A, POET Technologies Inc, Singapore, Singapore) broadband light source. A jumper wire was connected to one end of a 1 × 2 optical coupler, and the other end was connected to a superfluorescent fiber source with a spectrum analysis instrument. The reflected spectra of the FBG sensor were then scanned and stored via an optical spectrum analyzer (OSA, MS9740A, ANRITSU EMEA Ltd, Luton, UK). 

The TiN-coated FBG sensor (1551.00 nm 23 °C) was placed in the heating oven, which was certainly capable of influencing the data quality of the test experiments in terms of the signals emitted. The temperature of the oven was raised from 100 to 500 °C. Data were recorded once every 50 °C increase in temperature. Every individual temperature point was maintained for 10 min in order to record the reflective spectrum of the relationship between the wavelength and the temperature. 

### 3.2. Simulation of TiN-Coated FBG Sensor

#### 3.2.1. Thermal-Structure Interaction of TiN-Coated FBG

**Figure 5:**
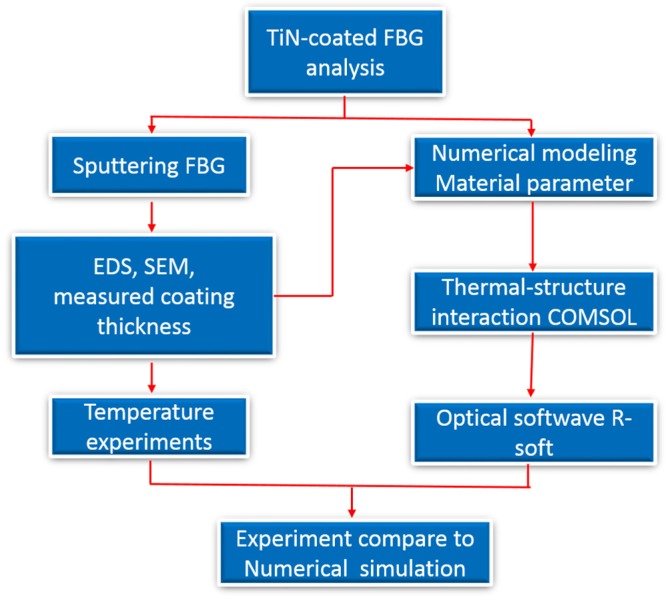
The schematic of the experimental design of the simulation study.

The design of the optical fiber temperature sensor was proven to be reasonable by the experimental results. The schematic of the experimental design is shown in Figure 5. The COMSOL Multiphysics 5.1 (v5.1.0.180, Pitotech Co. Ltd, Changhua, Taiwan) and optical software Rsoft (v2015.09, Cybernet Systems Taiwan Co. Ltd, Hsinchu, Taiwan)were then used for a temperature test simulation, with the temperature variation ranging from 100 to 500 °C with steps of 50 °C. A complete mesh consisting of 50,742 domain elements, 14,798 boundary elements, and 1372 edge elements was computed, and exhibited complete convergence. The TiN films and optical fiber material properties are shown in Table 1 [17,18]. The heat transfer interface of the TiN-coated FBG at temperatures from 100 to 500 °C was calculated with the COMSOL software. A linear relationship was obtained between the temperature loading and strain of the TiN-coated FBG, with a thermal sensitivity of 2.31 × 10^−6^.

#### 3.2.2. Numerical Modeling of Spectral Amplitude Response in FBG

The Rsoft software was then employed to analyze the temperature sensitivity of the FBG at various surrounding temperatures. For experimentation, the Rsoft software was initially employed to confirm the temperature sensitivity values measured by the FBG for the various surrounding temperatures by comparing the FBG change results with the Bragg wavelength shifts at the various temperatures. The results were then used to analyze the thermal expansion coefficient and the thermo-optic coefficient of the FBG temperature sensor.

Default waveguide settings were set in order to produce a standard single mode fiber with a diameter of 9.6 μm, a cladding index of 1.466, and a core index of 1.474. This corresponded to the following CAD parameters (as shown in Table 2): index difference = 0.008, background index = 1.466, and component width = component height = 9.6 µm. A typical free-space wavelength of 1551.78 nm was used at 100 °C, while the simulation test temperature ranged from 100 to 500 °C. The grating perturbation, defined as the way the waveguide parameters vary along the propagation direction, was 0.0003. The thermo-optic coefficient *ξ* and thermal expansion coefficient *α* of the fiber were defined as Dn/dt and DΛ/dt in the RSoft CAD layout parameters, and the simulation was performed as GratingMOD.

## 4. Results and Discussion

### 4.1. Temperature Test of the TiN-Coated FBG Sensor

**Figure 6:**
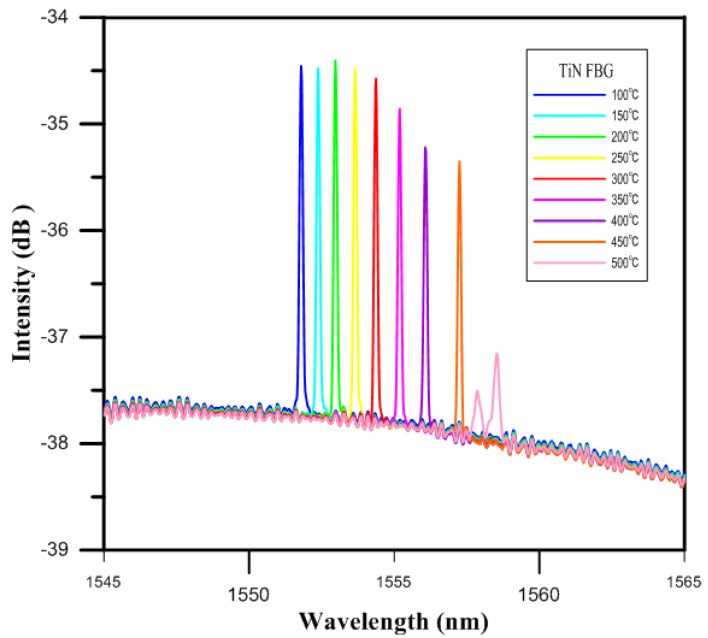
The online monitoring diagram of the highly reflective TiN-coated FBG.

The temperature sensitivity and responsivity of the TiN-coated FBG sensor in the oven was measured as light was injected into its fiber core by the broadband laser. As the test proceeded, the thermal expansion coefficient and refractive index in the grating of the TiN-coated FBG sensor changed. The changes in the temperature itself, meanwhile, could be observed via the reflection spectra of the FBG, which were themselves the result of perturbations in the grating resulting in shifts in the Bragg wavelength. Figure 6 shows the intensity spectra results of TiN-coated FBG as the temperature was increased.

**Figure 7:**
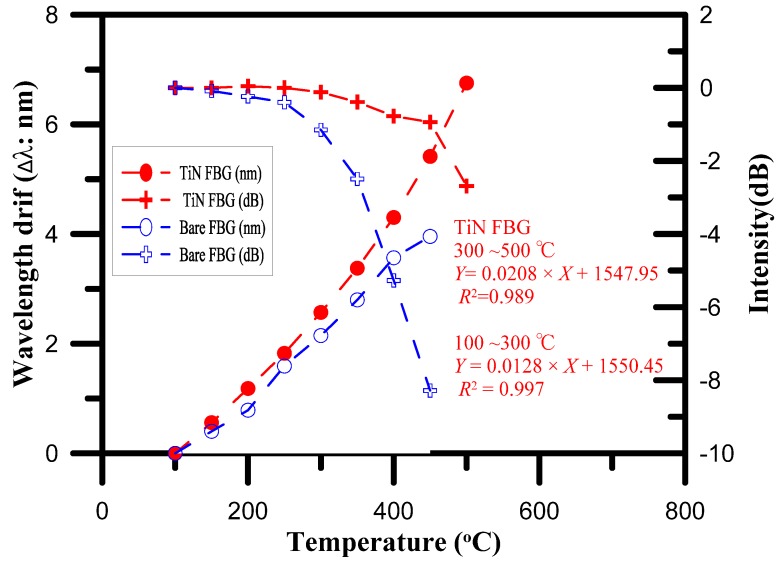
Temperature characteristics of the TiN-coated FBG sensor and bare FBG sensor.

The temperature characteristics of the TiN-coated FBG sensor showed that, under increasing temperature, the intensity spectrum of the TiN-coated FBG at 100 °C was measured to be 1551.78 nm, as shown in Figure 7. The results also showed that the shift in the Bragg wavelength for the TiN-coated FBG was 2.57 nm over the temperature range from 100 to 300 °C, while it was 4.18 nm for the higher temperature range from 300 to 500 °C.

Specifically, the responses of the TiN-coated FBG in terms of the shift in the Bragg wavelength for each degree of change in the temperature were calculated. The responsivity of the TiN-coated FBG was about 12.8 pm/°C for the temperatures from 100 to 300 °C, while it was about 20.8 pm/°C for the higher range of temperatures from 300 to 500 °C. Below 300 °C, the temperature coefficient of the TiN-coated FBG exhibited a 7.6% improvement over that of the bare FBG (|11.9–12.8|/11.9) [9].

**Figure 8:**
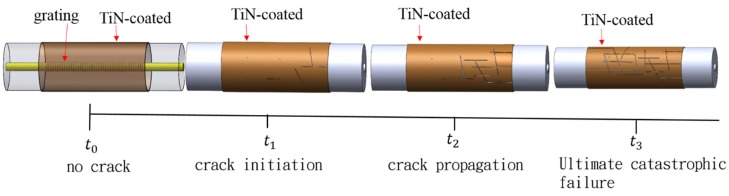
Schematic of crack characteristics of the TiN-coated FBG. (t_0~_t_3_ ≈ 90 s at 500 °C).

**Figure 9:**
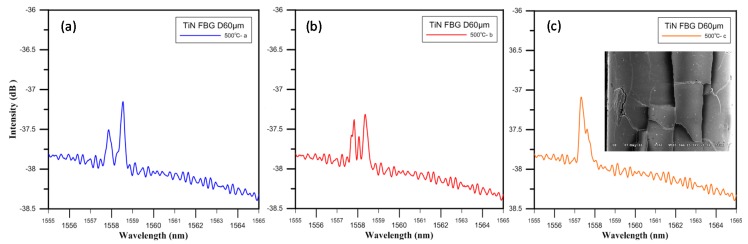
The optical spectra and SEM images of TiN-coated FBG at 500 °C: (**a**) crack initiation; (**b**) crack propagation, and (**c**) ultimate catastrophic failure.

The onset of oxidation occurred at 400 to 500 °C, and a change in the mechanism of TiN oxidation was observed with increasing temperature. Rapid oxidation occurred at 500 °C, and with increasing oxidation time, the growth and strain of the small crystallites arising from the volume expansion caused intergranular fracture. The oxidation crack propagation characteristics of the TiN-coated FBG at 500 °C, with t0~t3 ≈ 90 s, are schematically illustrated in Figure 8, where t1 indicates the crack initiation phase, t2 indicates the crack propagation phase, and t3 indicates the ultimate catastrophic failure phase. Optical spectra and SEM images of the experimental results for these different phases of oxidation time in the oven are shown in Figure 9. The crystallographic characteristics and intensity spectrum of the TiN-coated FBG film showed that oxidation crack propagation occurred at the maximum temperature of 500 °C. The spectra of the TiN-coated FBG exhibited two peaks (at 1557.86 nm and 1559.12 nm) for two different periods of crack initiation, three peaks (at 1557.80 nm, 1558.06 nm, and 1558.34 nm) for three different periods of crack propagation, and then one peak (at 1557.32 nm) for one period of ultimate catastrophic failure.

### 4.2. Analysis Using the FEM

**Figure 10:**
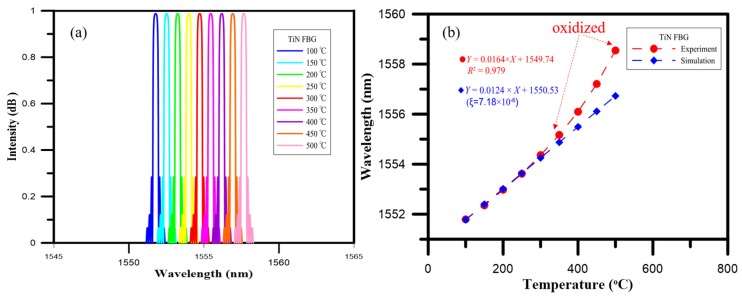
Temperature characteristics of the TiN-coated FBG sensor according to the Rsoft simulation and experiment. (**a**) Intensity-Wavelength graph for Rsoft simulation; (**b**) Wavelength-Temperature graph.

The temperature characteristics of the TiN-coated FBG sensor indicated by the Rsoft simulation and experiment are shown in Figure 10. The values measured by the TiN-coated FBG temperature sensor were for loadings of 0 to 500 °C, and were verified by experimental measurement. As can be seen from Figure 10a,b, the wavelength strength variation was 4.30 nm, and the sensitivity of this temperature sensor was 14.2 pm/°C for the experimental measurements, the wavelength variation of 3.71 nm and the sensitivity of 12.8 pm/°C, as indicated by the Rsoft software simulation analysis, were proportional to the temperature changes from 100 to 400 °C. These experimental results indicate that the Bragg wavelength shift of the TiN-coated FBG as the temperature changed was a parabolic relation, caused by the oxygen in the natural environment diffusing into the TiN-coated films and then diffusing through the grain boundary of the TiN-coated films into the SiO_2_, at temperatures from 150 to 400 °C. Additionally, a large part of the wavelength variation was observed at temperatures from 400 to 500 °C, where the temperature sensitivity (24.5 pm/°C) for the experiment measurement was higher than the simulation result, as oxidation occurred at that temperature range with formation of oxide layer of rutile-TiO_2_ [19].

## 5. Conclusions

This study proposed and investigated TiN-coated FBG applied as a sensor to monitor environmental temperatures. The sensor was fabricated by depositing a TiN film on FBG using PVD method. Simulations of this TiN-coated FBG were then conducted using the COMSOL and optical Rsoft software in order to obtain the temperature sensitivity, responsivity of the reflected spectrum, thermal expansion coefficient, and thermo-optic coefficient of the FBG temperature sensor. The experimental results indicated that the metal film of the TiN-coated FBG sensor had suitable temperature sensitivity and behavioral processes for the monitoring of crack growth, but it was found that the nonlinear part generated by the oxidation phenomenon could not be analyzed using the simulation software. This aspect is worthy of further investigation in an in-depth study. Moreover, the results indicated that the TiN-coated FBG sensor effectively achieved temperature sensitivity and responsivity for higher-temperature sensing at temperature ranges from 100 to 400 °C, with the sensitivity of the temperature sensor being 14.2 pm/°C for the experimental measurements; at temperatures from 400 to 500 °C, the temperature sensitivity 24.5 pm/°C for the experiment measurement.

## Figures and Tables

**Table 1:** TiN films and optical fiber material properties table [17,18].

Material	Elastic	Poisson’s	Coefficient of Thermal Expansion
TiN films	390 GPa	0.25	5.85 × 10^−6^
FBG	73 GPa	0.165	0.55 × 10^−6^

**Table 2:** The simulation parameters of TiN-coated FBG.

Simulation Tool	GratingMOD	Simulation Tool	GratingMOD
Grating type	Volume index	Width	9.6 µm
Structure type	Fiber	ModDelta	0.0003
Index profile	Step index	Delta	0.008
Length	5000 µm	Dn/dt (ξ)	7.18 × 10^−6^
Height	9.6 µm	DΛ/dt (α)	2.31 × 10^−6^

## References

[1] Sias F.R. (1957). A resistance-temperature detector for nuclear reactor service. Trans. Am. Inst. Electr. Eng. Part I Commun. Electron..

[2] Montalvo C., García-Berrocal A. (2015). Improving the in situ measurement of RTD response times through discrete wavelet transform in NPP. Ann. Nucl. Energy.

[3] Nicholas J.V., White D.R. (2001). Traceable Temperatures: An Introduction to Temperature Measurement and Calibration.

[4] Bogdan M., Vinţan M. (2003). Selecting the Right Sensor for Temperature Measurement.

[5] Hsieh T.-S., Chiang C.-C., Lin C.-H. (2014). A composite material leaf spring with fiber Bragg Grating as a sensor system for dynamic vehicle loading. Sens. Mater..

[6] Chiang C.-C., Chang H.-J., Kuo J.-S. (2010). Novel fabrication method of corrugated long-period fiber gratings by thick SU-8 photoresist and wet-etching technique. J. Micro/Nanolithogr. MEMS MOEMs.

[7] Mihailov S.J. (2012). Fiber Bragg Grating sensors for harsh environments. Sensors.

[8] Rajini-Kumar R., Suesser M.K., Narayankhedkar G., Krieg G., Atrey M.D. (2008). Performance evaluation of metal-coated fiber Bragg Grating sensors for sensing cryogenic temperature. Cryogenics.

[9] Hsiao T.-C., Hsieh T.-S., Chen Y.-C., Huang S.-C., Chiang C.-C. (2016). Metal-coated fiber Bragg Grating for dynamic temperature sensor. Optik.

[10] Zhang B., Kahrizi M. (2003). High-temperature Bragg Grating waveguide sensor. Proceedings of the International Conference on MEMS, NANO and Smart Systems.

[11] Zhang B., Kahrizi M. (2007). High-temperature resistance fiber Bragg Grating temperature sensor fabrication. IEEE Sens. J..

[12] Sharma R., Rohilla R., Sharma M., Manjunath D.T.C. (2005). Design & simulation of optical fiber Bragg Grating pressure sensor for minimum attenuation criteria. JATIT.

[13] Agarwal S., Mishra V. (2014). Characterization of fiber Bragg Grating for maximum reflectivity based on modulation depth of refractive index. Optik.

[14] Mishra V., Lohar M., Amphawan A. (2016). Improvement in temperature sensitivity of FBG by coating of different materials. Optik.

[15] Hsieh T.-S., Chen Y.-C., Chiang C.-C. (2016). Analysis and optimization of thermodiffusion of an FBG sensor in the gas nitriding process. Micromachines.

[16] Bai W., Yang M., Hu C., Dai J., Zhong X., Huang S., Wang G. (2017). Ultra-weak fiber Bragg Grating sensing network coated with sensitive material for multi-parameter measurements. Sensors.

[17] Mayrhofer P.H., Mitterer C., Hultman L., Clemens H. (2006). Microstructural design of hard coatings. Prog. Mater. Sci..

[18] Kashyap R. (2010). Principles of Optical Fiber Grating Sensors.

[19] Chen H.-Y., Lu F.-H. (2005). Oxidation behavior of titanium nitride films. J. Vac. Sci. Technol. A.

